# A Systematic Analytic Approach to Pandemic Influenza Preparedness Planning

**DOI:** 10.1371/journal.pmed.0020359

**Published:** 2005-11-01

**Authors:** Daniel J Barnett, Ran D Balicer, Daniel R Lucey, George S Everly, Saad B Omer, Mark C Steinhoff, Itamar Grotto

## Abstract

The World Health Organization warns that a flu pandemic is inevitable, and possibly imminent. Barnett and colleagues discuss a tool called the Haddon Matrix that could help in pandemic influenza planning.

The prospect of a pandemic with avian influenza is an urgent concern for public health leaders worldwide [1]. As pathogenic avian influenza A (H5N1) strains (Figure 1) continue to spread in East Asia, with recently reported expansion to Siberia and westward regions in Russia [2,3] as well as to migratory birds [4,5], the risk for reassortment of avian and human strains increases. Evidence cited by the World Health Organization in May 2005 suggests that H5N1 may be adapting to humans, thus potentially setting the stage for the next influenza pandemic [6].

**Figure 1:**
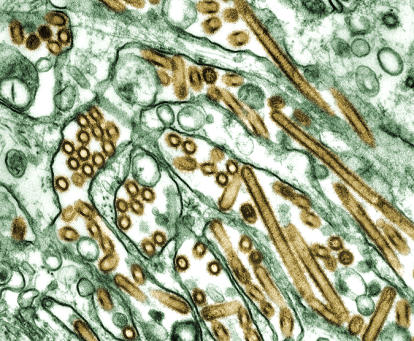
Colorized Transmission Electron Micrograph of Avian Influenza A H5N1 Viruses Grown in MDCK Cells The viruses are gold, and the MDCK cells are green. (Photo: CDC/C. Goldsmith, J. Katz, and S. Zaki)

Animal data suggest that the current H5N1 strain appears to be even more deadly than the original 1997 Hong Kong avian influenza, a finding that correlates well with the observed human case fatality rates [7]. As of August 5, 2005, there have been 112 human cases of H5N1 in East Asia resulting in 57 deaths (case fatality rate = 51%) [8,9]. Also concerning are recent findings that in China and Indonesia the virus has infected pigs, a possible “mixing vessel” for both avian and human influenza viruses, thus providing an opportunity for reassortment from which a pandemic human strain could emerge [10,11]. Research suggesting that cats could host or transmit the H5N1 infection [12] adds to a worrisome picture of multispecies transmission that can elevate the risk of reassortment [9]. This epizootic outbreak in Asia is not expected to wane in the short term [9].

Influenza pandemics can have devastating impacts. The Spanish flu of 1918 was particularly destructive (Figures 2 and 3), resulting in a higher death total in less than two years than in all of World War I [13]. Although earlier accounts suggested the mortality from the 1918 pandemic was 20 million to 40 million, more recent assessments including new estimates from Africa and Asia suggest that a more realistic figure is 50–100 million [14]. The high rates of infection with the pandemic virus meant that even an average case fatality rate lower than 3% resulted in this large number of deaths [13,15]. A 1918-type influenza pandemic today is projected to cause 180–360 million deaths globally (including 1.7 million deaths in the United States) [1], with transmission of the disease lasting at least two years [16].

**Figure 2:**
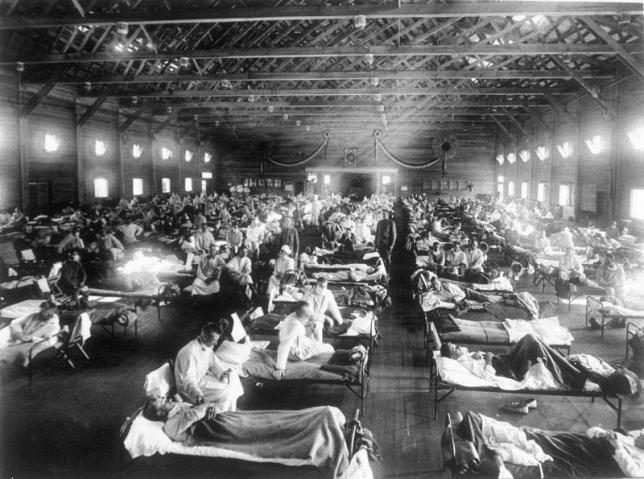
Emergency Hospital during 1918 Influenza Epidemic, Camp Funston, Kansas, United States (Photo: Image “NCP 1603,” National Museum of Health and Medicine, Armed Forces Institute of Pathology, Washington, D.C.)

**Figure 3:**
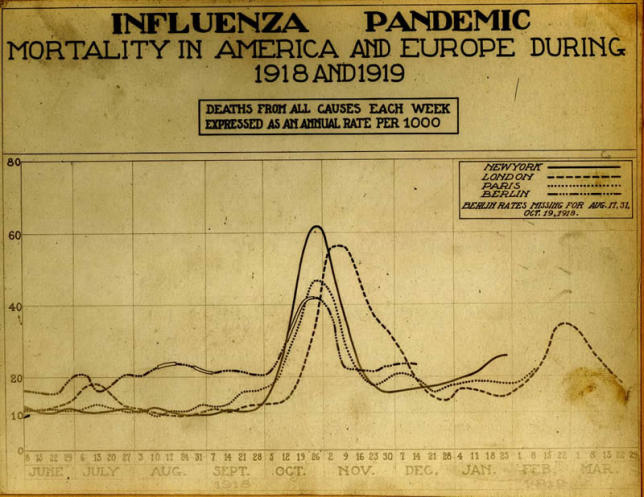
Historic Chart Showing Mortality Rates in America and Europe during 1918 and 1919 (Photo: Image “Reeve 3143,” National Museum of Health and Medicine, Armed Forces Institute of Pathology, Washington, D.C.)

## The Next Pandemic: “Inevitable, and Possibly Imminent”

In light of recent episodes of human infection with H5N1 virus, the World Health Organization reiterated its 1997 call for all countries to prepare for the next pandemic, which it termed “inevitable, and possibly imminent” [17], and updated its own pandemic plan in April 2005 [18]. In the United States, it has been argued that of the 12 disaster scenarios recently assessed by the US Department of Homeland Security, pandemic influenza is the most likely and perhaps the most deadly [19]. A draft form of the US pandemic influenza plan was made public in August 2004 [20], and an updated plan is anticipated by September 2005.

The urgent need for comprehensive pandemic influenza planning is profound: an influenza pandemic starting today may have major international consequences, including global economic and political destabilization, an overwhelming of health care resources, and panic [21]. Current international plans [18,22], while useful, could benefit from enhanced detail [21] and organization; moreover, pandemic influenza plans have usually been national in scope and, in most countries, are only in a draft form and lack legal status [23].

## The Haddon Matrix

An analytic approach for traffic safety injury epidemiology and prevention was developed by Dr. William Haddon, Jr. in the 1960s [24], and has since been termed “the Haddon matrix.” This matrix provides a multidimensional approach to understanding the contributing factors to injury before, during, and after an event [25]. The current version of the matrix is a grid with four columns, or axes, that represent contributing factors to injury (host, agent/vector, physical environment, sociocultural environment) and three rows that correspond to the time phases of a given form of injury (pre-event, event, and post-event) [26]. By compartmentalizing an injury into dimensions of time and contributing factors, the matrix can break a complex problem into more manageable segments. For each of the 12 cells, a decision analysis or prioritization can be used to select policies or actions with greatest feasibility or influence [27].

Although the Haddon matrix may seem unfamiliar to some infectious disease scientists, it incorporates familiar analytic elements in a systematic way. The four columns represent the classical epidemiologic triad of host, agent, and environment (physical and sociocultural). The three rows are equivalent to primary, secondary, and tertiary prevention of disease outbreaks. Indeed, Haddon himself used his analytic matrix to describe an outbreak of polio [24], and this matrix has been recently applied to other public-health emergency preparedness challenges such as SARS [28].

## Applying the Matrix to Pandemic Influenza Preparedness

Comprehensive public health emergency preparedness and response efforts require effective pre-event (preventive), event (mitigation), and post-event (consequence management) strategies. By identifying the factors that may modify the outcome in each of these phases, one can prescribe the appropriate measures necessary to tackle each factor.

To this end, we specifically applied the Haddon matrix to pandemic influenza planning and response (Table 1), systematically identifying relevant factors in each phase (pre-event, event, post-event) and on each axis (human, agent/vector, physical environment, sociocultural environment). We then identified factors that may be associated with opportunities for public health intervention, and marked these factors in bold within the matrix (consistent with an approach described by Runyan [27]).

**Table 1:**
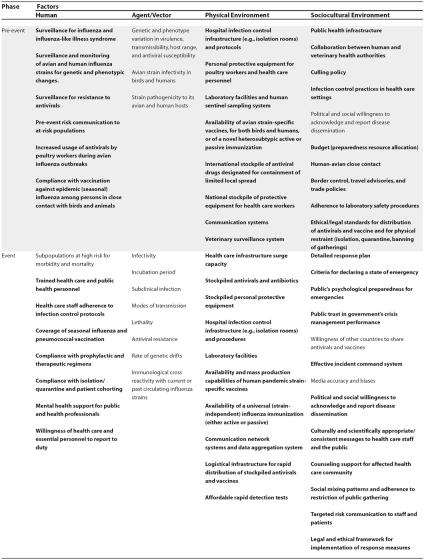
The Haddon Matrix and Pandemic Avian Influenza

Items in bold are potential targets for public health intervention.

**Table 1:**
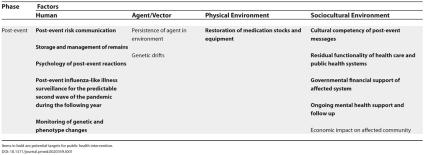
Continued

The table shows that in all phases of an influenza pandemic, opportunities for public health intervention include a number of contributing human, physical environment, and sociocultural factors, but generally not agent/vector factors, since viruses generally cannot be modified easily as injury-causing devices. Importantly, the pre-event, event, and post-event rows of the matrix reflect the phase of a pandemic in which public health preparedness and response measures will take their effects; however, planning for each of these measures must occur before the pandemic begins.

The use of the Haddon matrix in the table as an analytic and planning tool for pandemic influenza is illustrated below by its application to readiness efforts in two different countries: Thailand, focusing on pre-event factors; and Israel, focusing on event factors. We chose Thailand as an example because of its regional susceptibility and the proactive nature of its anti-H5N1 planning efforts to date. We selected Israel as an example of a country outside of East Asia that has taken steps to address this potential global crisis. For both countries, we demonstrate the application of the matrix by addressing selected factors within each axis.

## Pandemic Influenza Planning in Thailand

Thailand has had experience with H5N1 infections in both humans and animal populations—including chickens, ducks, birds, fighting roosters, and tigers—since January 2004. By October 2004, a total of 17 patients with H5N1 infection were identified, of whom 12 had died. The initial success of Thailand's national program against H5N1 avian influenza that began in the autumn of 2004 is evidenced by the fact that no human cases have been found between October 2004 and August 2005. Thus, Thailand's experience may offer practical lessons in preparing for an avian influenza-related human pandemic.

Through the lens of pre-event Haddon matrix factors, one can identify the strengths in Thailand's preparedness efforts, as well as opportunities for further enhancements. Selected examples of the pre-event axes for Thailand's pandemic influenza readiness efforts are described below.

### Pre-event human factors

Thailand has developed surveillance and laboratory testing algorithms for influenza-like-illness in humans and animals, including definitions for “suspect,” “probable,” “confirmed,” “excluded,” and “on investigation” cases of H5N1. With written guidance from national authorities [29], public health workers, veterinary health workers, village health volunteers, and others [30] participated in an ongoing surveillance campaign nationwide beginning in October 2004 [31].

Pre-event risk communication to at-risk populations are also important. In the scenario of pandemic influenza, effective pre-event risk communication can reduce event-phase risk communication barriers [32]. An array of appropriate information on avian influenza and potential pandemic human influenza has been disseminated by the Thai Ministry of Public Health [33].

### Pre-event agent/vector factors

Strain pathogenicity to its avian and human hosts is the major pre-event agent/vector factor. Most cases of human H5N1 infection have resulted from contact with infected chickens, fighting roosters, or ducks [9], with some ducks possibly being asymptomatic [34]. Regarding human pathogenicity, an autopsy of a patient from Thailand, one of the few involving H5N1 infection [35,36] reported that the virus can replicate in the human intestine as well as the lung [37] perhaps helping to explain the finding of diarrhea in some patients in Thailand and Vietnam [37–41].

### Pre-event physical environment factors

Thailand has established a multifaceted communication system, including websites for human and animal-related H5N1 updates and standard protocols. Provincial health offices were directed by the Ministry of Public Health to form Surveillance and Rapid Response Teams at the provincial and district levels [42].

Hospital infection control infrastructure and protocols are also crucial. Patients meeting criteria for possible H5N1 infection “should be isolated and placed in a single room according to the standard precautions of the Ministry of Public Health” [42]. Even if the patient's initial rapid test for influenza A is negative “the patient must be treated with antivirals immediately” [42] in an effort to increase survival [41].

The availability of avian strain-specific vaccines is another significant factor. Webster and Hulse observed that Thailand's investigation of flu vaccines for “open range” (noncommercial) poultry represents a “prudent” policy shift that should be replicated in other countries in East Asia [43]. H5N1 vaccine studies in humans have not yet been initiated in Thailand.

### Pre-event sociocultural factors

One of the most significant factors is political and social willingness to acknowledge and report disease dissemination. Initially, the Thai government was criticized for underplaying the existence and the magnitude of avian influenza in Thailand [44] but it has since taken significant proactive steps to address this urgent challenge. Between January 2004 and July 2005, a total of 59 official reports on surveillance for Highly Pathogenic Avian Influenza have been submitted to the World Organization for Animal Health by Thailand [45], and detailed reports were promptly published [35,36,38,41,46–48]. On September 28, 2004, the first media report of a probable case of person-to-person transmission appeared in Thailand [49] and was rapidly published [36]. On September 29 of that year, a national campaign against the H5N1 virus was declared by the Prime Minister of Thailand, with involvement by the Thai Cabinet [50,51]. These resulting efforts seem to have had a substantial impact, as detailed above.

Budget (preparedness resource allocation) is also important. The Thai National Strategic Plan for Avian Influenza and Plan for Pandemic Preparedness 2005–2007 was initiated with a budget of 4,026 million Thai baht (∼US$105 million) [52,53]. Thailand has been reported recently to have approved funding for the future purchase of up to 100,000 treatments of oseltamivir [54].

An in-place culling policy played a significant role. The culling of ducks (with farmer compensation) reduced the flocks that were positive for H5N1 from around 40% infected in October 2004 to almost undetectable levels in March 2005 [43].

Collaboration between human and veterinary health authorities was vital. Efforts are ongoing to closely link public health and animal health responses to H5N1 [52,53]. Surveillance combines epidemiologically linked testing for animals and humans [55]. In addition, Thailand interacts frequently with the World Health Organization regarding clinical H5N1 issues, and with the World Organization for Animal Health in reporting on animal surveillance for H5N1 [56].

## Pandemic Influenza Planning in Israel

Applying the various influencing factors listed in the event phase of the Haddon matrix to the unique Israeli setting leads to several important insights regarding local pandemic preparedness, as shown in the following examples.

### Event human factors

Israel has not initiated, as of yet, training activities for health care professionals directed specifically at pandemic preparedness, although such activities are planned to take place. Nevertheless, Israeli health care professionals, particularly frontline health care workers, are well experienced with terrorism-related mass casualty emergencies. Continuous training of the various components of the health care system for bioterrorism threats likely serves to enhance these workers' ability to deal with naturally occurring epidemic threats; these health care teams were shown to have increased likelihood of reporting to duty during a crisis [57]. Simulation-assisted medical training may be useful in increasing health care workers' compliance with personal protective equipment and infection control protocols, as has been shown in the preparation of Israeli medical teams to respond to chemical warfare casualties [58]. Upcoming tabletop exercises will test and refine current national contingency plans, while full-scale drills may be required to test certain practical and logistical aspects of antiviral drug dissemination.

### Event agent/vector factors

Agent/vector factors listed in the matrix are expected to determine much of the local impact of the pandemic, but they generally cannot be influenced by preparedness and mitigation efforts. As these factors will remain unknown until the first stages of the pandemic, Israeli preparedness planners have taken into account a wide range of scenarios with different attack and mortality rates [59] in addressing issues such as surge capacity. For instance, a highly transmissible pandemic may render isolation and quarantine efforts largely futile [60] while a less transmissible strain, as witnessed in previous pandemics [61] may enable a containment approach more similar to that taken during the SARS epidemic (while accounting for considerable differences such as the incubation time or the impact of infectious asymptomatic cases). A highly pathogenic strain, perhaps more pathogenic than the 1918 strain (considering current case fatality rates of H5N1 human cases), will require the unparalleled ability to rapidly mobilize medical equipment and personnel to meet the increased demands for care in both primary and secondary care facilities. However, a less pathogenic strain may require measures similar to those taken during severe seasonal influenza epidemics.

### Event physical environment factors

The availability of an effective immunization will be crucial. The importance of the recently published successful preliminary results of phase-I human H5N1 vaccine trials cannot be overestimated [62]. Nevertheless, both the safety and efficacy of the new vaccine remain to be assessed, and the effectiveness of this vaccine against a reassortant pandemic strain is currently difficult to predict.

Research efforts to produce active or passive immunization that will be universally effective against any influenza strain are currently underway in Israel and elsewhere. Once available, such modalities hold great promise for mitigation of future pandemics in their first stages [63]. Another type of immunotherapy that may be considered during an event is the use of immunoglobulins isolated from recovered patients to treat the ill or protect the exposed.

Stockpiled antivirals and antibiotics are important to Israel's preparedness. The Israeli Ministry of Health has successfully used cost-benefit analyses [59] to persuade decision makers to invest the funds necessary for the rapid creation of a national antiviral stockpile, and several strategies for the use of these drugs during the pandemic are considered [64]. The antiviral oseltamivir was found to be effective in mice against the newest strains of avian influenza currently sweeping through East Asia, suggesting that higher doses and prolonged courses of this drug may be required [7]. These findings, if validated in humans, may need to be factored into stockpiling planning efforts.

### Event sociocultural factors

Israel has ensured that a legal and ethical framework for implementation of response measures exists. Including pandemic influenza in the list of “dangerous communicable diseases” defined by Israeli law will allow the Ministry of Health to uphold extreme measures such as involuntary quarantine and isolation, if needed.

Prioritizing target groups for antiviral drugs and vaccines, expected to be in short supply, requires the addressing of complex ethical, legal, social, and political considerations. The choice of which groups to prioritize would derive, in part, from the prioritizing of the various goals in using these drugs. If the focus is on reducing all mortality, different groups may be prioritized than if the main attempt is to reduce social disruption. A national ethics committee was recently appointed to address these issues.

## Conclusions

By offering phase-specific insights into pandemic influenza planning, the Haddon matrix bridges injury-prevention epidemiology with global infectious disease preparedness and response. In the process, this analytic tool sheds light on opportunities for prevention, mitigation, and consequence management strategies to address a global public health threat.

In the face of the challenges described, the Haddon matrix analysis of pandemic influenza planning in Thailand and Israel reflects its applicability as a systematic tool for identifying urgent national and international pandemic avian influenza readiness needs. The scalability of the matrix also allows its use at the level of a county or city, as well as within institutions. At each of these levels, the matrix may facilitate the enhancement of preparedness plans, needs assessments, best practice identification, and resource distribution strategies.

Although the national examples above have selectively focused on pre-event factors in Thailand and event factors in Israel, the Haddon matrix can be also used to augment existing post-event phase plans. For example, the psychology of post-event reactions [65] must be addressed through ongoing mental health support and follow up and by effective post-event risk communication. The public health infrastructure may face the dual challenge of helping populations, including health care providers themselves, to be psychologically prepared for the next wave of a pandemic—perhaps worse than the first wave, as was the case in the 1918 pandemic [13]—while trying to recover from the first wave.

The Haddon matrix has limitations that must be recognized to ensure appropriate implementation. Importantly, the matrix is not a stand-alone planning tool; rather, the results of any Haddon-matrix–based analysis must be operationalized in the form of policies and procedures to achieve their desired effects on the factors included in the matrix. Moreover, the matrix is not static; the contents within its cells can and should be modified according to changing disease dynamics and situational challenges to maintain its usefulness in an evolving crisis.

Furthermore, even before a crisis, the choice of contents for each cell is not absolute, and open to the subjective interpretation of those who are preparing the matrix. Consequently, the table presented in this article should be regarded as a starting planning framework, not a final checklist. Also, while many of the items in the Haddon matrix cells may be measurable, the matrix itself is only a planning instrument—not an evaluation tool.

The known potential for an avian influenza pandemic offers not only challenges but also unprecedented opportunities for advance planning at all levels of public health in the international community [66]. This planning window may be rapidly closing, however [21]. As an efficient yet comprehensive analytic approach, the Haddon matrix lends itself to the types of rapid and complex decision making necessary to plan for and respond more effectively to an urgent pandemic health threat.

## Abbreviation

H5N1: avian influenza A
